# Severe versus common COVID-19: an early warning nomogram model

**DOI:** 10.18632/aging.203832

**Published:** 2022-01-17

**Authors:** Yanxin Chang, Xuying Wan, Xiaohui Fu, Ziyu Yang, Zhijie Lu, Zhenmeng Wang, Li Fu, Lei Yin, Yongjie Zhang, Qian Zhang

**Affiliations:** 1 Biliary Tract Surgery Department IV, Eastern Hepatobiliary Surgery Hospital, Second Military Medical University, Shanghai 200438, PR China; 2 Infectious Disease Department IV, Hubei Maternal and Child Health Hospital, Wuhan 430074, PR China; 3 Department of Integrated Traditional and Western Medicine, Eastern Hepatobiliary Surgery Hospital, Second Military Medical University, Shanghai 200438, PR China; 4 Biliary Tract Surgery Department II, Eastern Hepatobiliary Surgery Hospital, Second Military Medical University, Shanghai 200438, PR China; 5 Infectious Disease Department II, Huoshenshan Hospital, Wuhan 430113, PR China; 6 Department of Anesthesiology, Eastern Hepatobiliary Surgery Hospital, Second Military Medical University, Shanghai 200438, PR China; 7 Department of Biotherapy, Eastern Hepatobiliary Surgery Hospital, Second Military Medical University, Shanghai 200438, PR China

**Keywords:** COVID-19, early warning nomogram model, severe versus common, risk factors, validation

## Abstract

The wide spread of coronavirus disease 2019 is currently the most rigorous health threat, and the clinical outcomes of severe patients are extremely poor. In this study, we establish an early warning nomogram model related to severe versus common COVID-19. A total of 1059 COVID-19 patients were analyzed in the primary cohort and divided into common and severe according to the guidelines on the Diagnosis and Treatment of COVID-19 by the National Health Commission of China (7th version). The clinical data were collected for logistic regression analysis to assess the risk factors for severe versus common type. Furthermore, 123 COVID-19 patients were reviewed as the validation cohort to assess the performance of this model. Multivariate logistic analysis revealed that age, dyspnea, lymphocyte count, C-reactive protein and interleukin-6 were independent factors for prewarning the severe type occurrence. Then, the early warning nomogram model including these risk factors for inferring the severe disease occurrence out of common type of COVID-19 was constructed. The C-index of this nomogram in the primary cohort was 0.863, 95% confidence interval (CI) (0.836–0.889). Meanwhile, in the validation cohort, the C-index of this nomogram was 0.889, 95% CI (0.828–0.950). In both the primary cohort and validation cohorts, the calibration curve showed good agreement between prediction and actual probability. The early warning model shows that data at the very beginning including age, dyspnea, lymphocyte count, CRP, and IL-6 may prewarn the severe disease occurrence to some extent, which could help clinicians early and timely treatment.

## INTRODUCTION

Coronaviruses are single-stranded, positive-sense RNAs consisting of four genera, including alpha-coronavirus (α-CoV), beta-coronavirus (β-CoV), delta-coronavirus (δ-CoV) and gamma-coronavirus (γ-CoV), which are viruses that have caused severe global infections, such as severe acute respiratory syndrome (SARS) and Middle East respiratory syndrome (MERS) [1, 2]. As a novel beta-coronavirus, severe acute respiratory syndrome coronavirus 2 (SARS-CoV-2) is characterized by an enveloped RNA virus and pleomorphic shape with a diameter of 60–140 nm [3]. It is known as the cause of coronavirus disease 2019 (COVID-19), a highly infectious disease that has been declared a global public health emergency by the World Health Organization (WHO). The COVID-19 outbreak has rapidly transitioned into a worldwide pandemic owing to the major transmission of respiratory droplets as well as contact transmission (such as inhalation of aerosols in certain activities (e.g., singing, intubation or the use of nebulizers) or even contamination of inanimate surfaces). Up to April 26, 2020, the COVID-19 pandemic caused more than 2,719,897 cases and 187,705 deaths around the world, with over 200 countries affected [4].

According to the latest version of the guidelines on the Diagnosis and Treatment of COVID-19 by the National Health Commission of China, patients infected with COVID-19 are clinically defined as mild, moderate, severe and critical [3]. Among them, mild patients present light symptoms without signs of pneumonia, and moderate patients start to show fever, respiratory symptoms and pneumonia by imaging. For severe patients, most of them develop dyspnea and/or hypoxia within one week, and even critical cases progress rapidly to acute respiratory distress symptom (ARDS), septic shock, irreversible metabolic acidosis, coagulation dysfunction and multi-organ dysfunction. Laboratory indexes show normal or reduced peripheral white blood cell (WBC) count, total lymphocyte count, increased erythrocyte sedimentation rate (ESR), and C-reactive protein (CRP) and partial reduction of heparinase (Hpa), lactate dehydrogenase (LDH), creatase, and myoglobin in moderate patients. Severe patients have decreased D-dimer and progressive reduction of peripheral lymphocytes or elevated inflammatory cytokines in severe and critical patients. At present, most patients are mild and moderate at diagnosis, but a few patients can progress rapidly to severe, critical or even death [3]. Since there is still no consensus on the factors that lead to the progression from common (mild and moderate) to severe (severe and critical) disease, the clinical diagnosis and treatment of COVID-19 is difficult. Therefore, an early warning consensus of severe patient is needed to guide early and timely treatment.

Inspired by the use of predictive models for prognosis in other diseases, we retrospectively reviewed the medical records of 1059 COVID-19 patients, conducted logistic regression analysis for factors related to severe versus common type and established an early warning nomogram model including these high risk factors of severe disease occurrence in COVID-19 patients. Then, the early warning nomogram model was validated in another cohort including 123 COVID-19 patients.

## RESULTS

### COVID-19 patient characteristics

In the primary cohort, a total of 1059 COVID-19 patients were enrolled, and there were 808 common patients (including 326 (40.3%) males and 482 (59.7%) females) and 251 severe patients (including 133 (53.0%) males and 118 (47.0%) females). In the validation cohort, a total of 123 COVID-19 patients were recruited, including 90 common patients (including 46 (51.1%) males and 44 (48.9%) females) and 33 severe patients (including 21 (63.6%) males and 12 (36.4%) females). The clinical characteristics and laboratory findings of COVID-19 patients in the primary and validation cohorts are listed in Table 1.

**Table 1:** Clinical characteristics and laboratory findings of patients with COVID-19 in primary cohort and validation cohort.

**Features**	**Primary cohort (*N* = 1059)**		**Validation cohort (*N* = 123)**	
	**Common type (*n* = 808)**	**Severe type (*n* = 251)**	**Common type (*n* = 90)**	**Severe type (*n* = 33)**
	**No. (%)**	**No. (%)**	**No. (%)**	**No. (%)**
**Sex**				
Male	326 (40.3)	133 (53.0)	46 (51.1)	21 (63.6)
Female	482 (59.7)	118 (47.0)	44 (48.9)	12 (36.4)
**Age, years**				
<60	439 (54.3)	58 (23.1)	66 (73.3)	6 (18.2)
≥60	369 (45.7)	193 (76.9)	24 (26.7)	27 (81.8)
**Weight, kg**				
<64	409 (50.6)	165 (65.7)	46 (51.1)	15 (45.5)
≥64	399 (49.4)	86 (34.3)	44 (48.9)	18 (54.5)
**Symptoms**				
Dyspnea	191 (23.6)	140 (55.8)	17 (18.9)	21 (63.6)
Fever	535 (66.2)	166 (66.1)	54 (60.0)	21 (63.6)
Cough	424 (52.5)	127 (50.6)	58 (64.4)	18 (54.5)
Fatigue	215 (26.6)	72 (28.7)	32 (35.6)	9 (27.3)
**Laboratory tests**				
Leukocyte count, ×10/L				
<9.5	781 (96.7)	217 (86.5)	88 (97.8)	32 (97.0)
≥9.5	27 (3.3)	34 (13.5)	2 (2.2)	1 (3.0)
Lymphocyte count, ×10/L				
<1.1	110 (13.6)	115 (45.8)	12 (13.3)	14 (42.4)
≥1.1	698 (86.4)	136 (54.2)	78 (86.7)	19 (57.6)
Neutrophil count, ×10/L				
<6.3	776 (96.0)	200 (79.7)	88 (97.8)	28 (84.8)
≥6.3	32 (4.0)	51 (20.3)	2 (2.2)	5 (15.2)
Monocytes count, ×10/L				
<0.6	759 (93.9)	230 (91.6)	85 (94.4)	32 (97.0)
≥0.6	49 (6.1)	21 (8.4)	5 (5.6)	1 (3.0)
CRP, mg/L				
<0.6	668 (82.7)	94 (37.5)	84 (93.3)	18 (54.5)
≥0.6	140 (17.3)	157 (62.5)	6 (6.7)	15 (45.5)
ALT, U/L				
<55	735 (91.0)	207 (82.5)	85 (94.4)	28 (84.8)
≥55	73 (9.0)	44 (17.5)	5 (5.6)	5 (15.2)
AST, U/L				
<34	752 (93.1)	208 (82.9)	86 (95.6)	24 (72.7)
≥34	56 (6.9)	43 (17.1)	4 (4.4)	9 (27.3)
ALP, U/L				
<79	600 (74.3)	147 (58.6)	73 (81.1)	20 (60.6)
≥79	208 (25.7)	104 (41.4)	17 (18.9)	13 (39.4)
TBIL, μmol/L				
<20.5	769 (95.2)	227 (90.4)	87 (96.7)	30 (90.9)
≥20.5	39 (4.8)	24 (9.6)	3 (3.3)	3 (9.1)
PCT, ng/ml				
<0.04	647 (80.1)	140 (55.8)	87 (96.7)	30 (90.9)
≥0.04	161 (19.9)	111 (44.2)	3 (3.3)	3 (9.1)
IL-6, pg/mL				
<10	781 (96.7)	169 (67.3)	87 (96.7)	20 (60.6)
≥10	27 (3.3)	82 (32.7)	3 (3.3)	13 (39.4)

Abbreviations: COVID-19: Corona Virus Disease 2019; CRP: C-reactive protein; ALT: alanine aminotransferase; AST: aspartate aminotransferase; ALP: Alkaline phosphatase; TBIL: total bilirubin; PCT: procalcitonin; IL-6: interleukin 6.

### Risk factors of the severe type of COVID-19

Univariate logistic regression analysis showed that age (≥60 years *V* < 60 years) (*P* < 0.001), dyspnea (yes *V* no) (*P* < 0.001), lymphocyte count (low *V* normal) (*P* = 0.001), CRP (high *V* normal) (*P* < 0.001) and IL-6 (high *V* normal) (*P* < 0.001) were correlated with the occurrence of severe type out of the common type of COVID-19 (Table 2).

**Table 2:** Univariate logistic regression analysis of factors related to the progression from common type to severe type of COVID-19.

**Variables**	**Univariate logistic regression model**				
	**B**	**S.E.**	***P* value**	**OR**	**95% CI**
Sex (male *V* female)	0.218	0.197	0.268	1.243	0.846–1.828
Age (≥60 years *V* < 60 years)	1.173	0.198	<0.001	3.233	2.193–4.765
Weight (≥64 kg *V* < 64 kg)	0.215	0.201	0.285	1.239	0.837–1.836
Dyspnea (yes *V* no)	1.325	0.189	<0.001	3.761	2.599–5.443
Fever (yes *V* no)	−0.180	0.195	0.355	0.835	0.570–1.223
Cough (yes *V* no)	−0.104	0.183	0.572	0.901	0.629–1.292
Fatigue (yes *V* no)	−0.123	0.210	0.559	0.884	0.586–1.335
Leukocyte count (high *V* normal)	−0.291	0.545	0.593	0.747	0.257–2.175
Lymphocyte count (low *V* normal)	0.719	0.212	0.001	2.052	1.355–3.108
Neutrophil count (high *V* normal)	0.790	0.480	0.100	2.203	0.859–5.645
Monocytes count (high *V* normal)	−0.577	0.363	0.112	0.562	0.275–1.145
CRP (high *V* normal)	1.242	0.215	<0.001	3.464	2.274–5.274
ALT (high *V* normal)	0.514	0.335	0.124	1.673	0.868–3.223
AST (high *V* normal)	−0.303	0.362	0.402	0.738	0.363–1.502
ALP (high *V* normal)	0.050	0.204	0.806	1.051	0.705–1.567
TBIL (high *V* normal)	−0.049	0.385	0.898	0.952	0.447–2.025
PCT (high *V* normal)	0.345	0.205	0.092	1.412	0.945–2.111
IL-6 (high *V* normal)	1.376	0.303	<0.001	3.960	2.186–7.171

Abbreviations: COVID-19: Corona Virus Disease 2019; B: regression coefficient; S.E.: standard error; OR: odds ratio; CI: confidence interval; CRP: C-reactive protein; ALT: alanine aminotransferase; AST: aspartate aminotransferase; ALP: Alkaline phosphatase; TBIL: total bilirubin; PCT: procalcitonin; IL-6: interleukin 6.

### Independent factors for prewarning the severe type of COVID-19

Multivariate logistic regression analysis disclosed that age (≥60 years *V* < 60 years) (*P* < 0.001), dyspnea (yes *V* no) (*P* < 0.001), lymphocyte count (low *V* normal) (*P* < 0.001), CRP (high *V* normal) (*P* < 0.001) and IL-6 (high *V* normal) (*P* < 0.001) were independent factors in prewarning the severe disease occurrence of COVID-19 (Table 3), which may help clinicians early and timely treatment.

**Table 3:** Multivariate logistic regression analysis of factors related to the progression from common type to severe type of COVID-19.

**Variables**	**Multivariate logistic regression model**				
	**B**	**S.E.**	***P* value**	**OR**	**95% CI**
Age (≥60 years *V* < 60 years)	1.124	0.193	<0.001	3.078	2.107–4.496
Dyspnea (yes *V* no)	1.336	0.181	<0.001	3.802	2.665–5.425
Lymphocyte count (low *V* normal)	0.842	0.202	<0.001	2.320	1.561–3.449
CRP (high *V* normal)	1.374	0.199	<0.001	3.953	2.676–5.839
IL-6 (high *V* normal)	1.451	0.285	<0.001	4.267	2.443–7.453

Abbreviations: COVID-19: Corona Virus Disease 2019; B: regression coefficient; S.E.: standard error; OR: odds ratio; CI: confidence interval; CRP: C-reactive protein; IL-6: interleukin 6.

### Early warning nomogram construction for severe type occurrence

**Figure 1:**
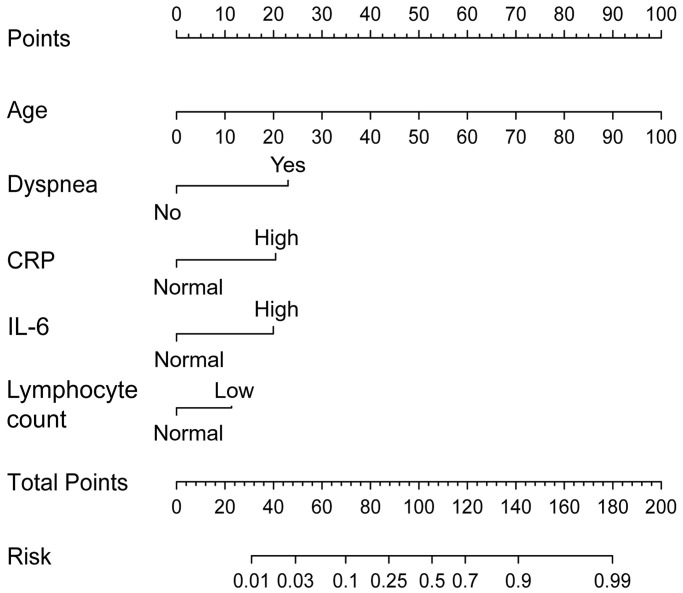
**Risk prewarning nomogram for severe type patients.** Each patient’s variables could be located on the corresponding variable axis. The point of each variable could be determined by vertically referring to the top point line. By summing up the total points of each corresponding variables, total point was calculated, and risk of disease progression was determined by reading against the risk axis.

**Figure 2:**
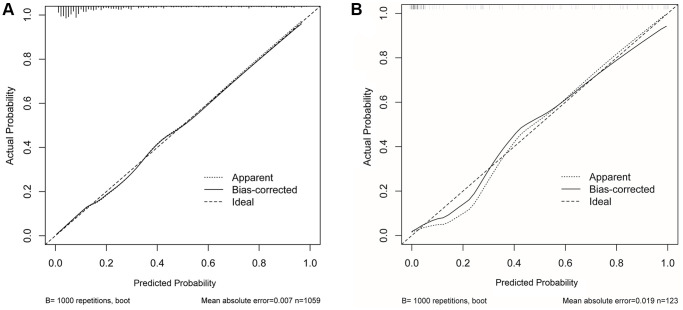
**The calibration curves of nomogram in prewarning the severe infection occurrence**. Nomogram predicted severe type risk was plotted on x-axis, the actual disease progression probability was plotted on y axis. (**A**) Training cohort; (**B**) Validation cohort.

**Figure 3:**
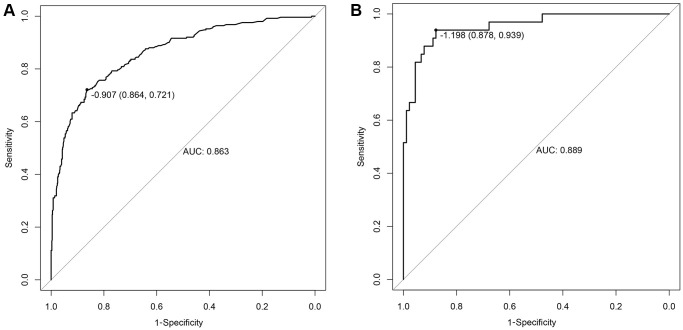
**The ROC curves of the nomogram.** (**A**) Training cohort; (**B**) Validation cohort.

The early warning nomogram was constructed based on the independent factors for inferring the severe disease occurrence out of common type of COVID-19. As shown in Figure 1, each variable could be assessed vertically to obtain the point, and the total points of all five variables were calibrated correspondingly to the risk. Age was the strongest contributor to the progression from common type to severe type COVID-19, followed by dyspnea, CRP, IL-6 and lymphocyte count. The C-index of this nomogram was 0.863, 95% CI (0.836–0.889). In addition, the early warning accuracy of this nomogram in the primary cohort is illustrated in Figure 2A, the calibration curve showed excellent agreement between prediction and actual probability. Also, the receiver operating characteristic (ROC) curve was employed to assess the predictive ability of the established nomogram, and the area under curve (AUC) was 0.863 in the training cohort, with a sensitivity of 72.1% and specificity of 86.4% (*P* < 0.001, Figure 3A).

### Validation of the early warning accuracy of the nomogram for disease progression

The accuracy of the nomogram for disease progression was validated in another cohort including 123 patients. In the calibration curve of Figure 2B, the C-index of this nomogram in the validation cohort was 0.889, 95% CI (0.828–0.950). The calibration curve showed good agreement between the prediction and actual probability. Moreover, in the validation cohort, the AUC of ROC curve was 0.889 with a sensitivity of 93.9% and specificity of 87.8% (*P* < 0.001, Figure 3B). In both the primary cohort and validation cohort, the calibration plots presented good consistency between prediction and actual probability. Thus, the nomogram showed good accuracy and discrimination ability in early identifying the occurrence of severe disease out of common type of COVID-19.

### Clinical application of the early warning nomogram

**Figure 4:**
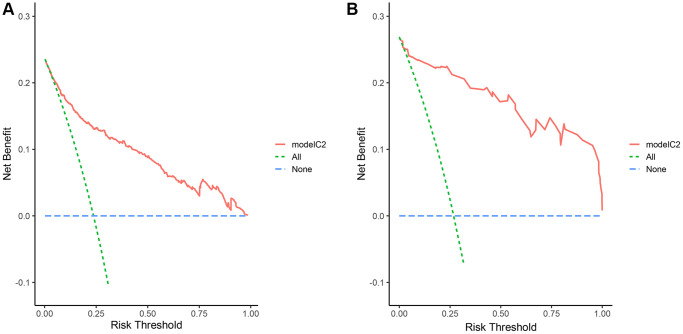
**DCA curves of the nomogram.** DCA compares the net benefits of three scenarios in prewarning the severe disease occurrence: A perfect prediction model (blue line), screen none (horizontal green line), and screen based on the nomogram (red line). The DCA curves were depicted in the training cohort (**A**), validation cohort (**B**).

In order to assess the clinical applicability of the risk prediction nomogram, Decision curve analysis (DCA) based on the net benefit and threshold probabilities was performed. As shown in Figure 4, whether in primary or validation cohort, our risk prediction nomogram had a superior net benefit with a wide range of threshold probabilities.

## DISCUSSION

There is a large difference in prognosis between common patients and severe patients. A study on COVID-19 patients from Jinyintan Hospital and Wuhan Pulmonary Hospital reported that severe and critical patients show significantly higher mortality than general patients [5]. The in-hospital observations also show high mortality in patients admitted to extracorporeal membrane oxygenation (ECMO) and a rapid drop in SaO in severe patients. Therefore, investigating the influential factors that underlie the transition of common to severe disease is extremely important for the early identification of patients at risk as well as time management. We believe that the progression of common to severe disease occurs earlier physiologically. For instance, some patients had sharp deterioration soon after slightly increased physical activity. This indicated that the disease had already progressed over the common threshold to severe before the patient started to show severe symptoms. As analyzed by existing evidence, there are risk factors for mortality in COVID-19 patients, including older age, high D-dimer level and high Sequential Organ Failure Assessment (SOFA) score at admission [5]. In addition, the signs for ICU admission, including dyspnea, low WBC count, high neutrophil count and D-dimer level, are also critical references for the diagnosis of severe disease [6]. According to these risk factors for ICU admission as well as mortality, it is presumable that analyzing patients’ characteristics at hospital admission could help with identifying common to severe progression and may aid appropriate supportive care and prompt access to the ICU if necessary. Considering the clinical feasibility, we comprehensively extracted key clinical features of 1059 COVID patients, including sex, age, weight, symptoms and laboratory tests, conducted logistic regression analysis and successfully constructed an early warning model for severe disease occurrence.

Herein, we discovered that age (≥60 years *V* < 60 years), dyspnea (yes *V* no), lymphocyte count (low *V* normal), CRP (high *V* normal) and IL-6 (high *V* normal) were independent factors to infer the severe disease occurrence out of common type of COVID-19, in which age was the most important contributor. In terms of age (≥60 years *V* < 60 years), older patients featured a poor physical state and decreased immune function, which made them vulnerable to infections and difficult to quickly relieve in the face of COVID-19, subsequently prolonging the disease course to progress rapidly to severe or critical or even death [7, 8]. In addition, older patients usually have several comorbidities (including hypertension [9], diabetes [10] and cardiovascular disease [11]. Considering that lung infections caused by this novel coronavirus might increase the burden on the heart and cause high blood sugar, the characteristics of multisystem disease coexisting in the elderly lead to complicated and complex diseases. Multiple diseases affect each other, and the difficulty of treatment is greatly increased. Thus, elderly patients are prone to multisystem organ dysfunction and even failure, thereby progressing rapidly to severe or critical or even death [7]. Older age could mostly infer the severe disease occurrence out of common type of COVID-19.

Regarding dyspnea (yes *V* no), SARS-CoV-2 entered the lungs via respiration and then activated immune cells, cytokines and other pathogen-resistance systems [12]. With an increase in virus virulence, patient immunity might decrease during the struggle between the virus and the human host, which might result in congestion and edema in lung tissue, thickening of the interstitial lung and increased exudation in the alveolar space to form a transparent membrane-like structure [12]. These severe pathological changes in lung tissue might directly make patients feel dyspnea. Hence, dyspnea could be an independent factor to prewarn the severe disease occurrence out of common type of COVID-19.

In terms of lymphocyte count (low *V* normal), after SARS-CoV-2 infection, lymphopenia is a typical laboratory abnormality, which is similar to other highly pathogenic coronavirus infections (including SARS-CoV [13] and MERS-CoV [14] infections). Lymphocyte subsets play an important role in cellular immune regulation, and SARS-CoV-2 infection has been reported to primarily impact T lymphocytes (especially CD4+ T and CD8+ T cells), which means that T lymphocytes might be highly involved in the pathological process of COVID-19 and provide an important defense against COVID-19 [12, 15]. Additionally, its magnitude of decrease suggested the extent of impairment of the immune system by viral infection [12, 16]. Thus, lymphocyte count might act as a powerful factor for independently indicating the severe disease occurrence out of common type of COVID-19.

CRP and IL-6, two common proinflammatory factors, have been confirmed to be associated with severe lung injury and adverse clinical outcomes of SARS-CoV or MERS-CoV infection [14, 17–19]. After SARS-CoV-2 infection, CRP and IL-6 also increased rapidly in a short time [12], which is in line with the concept of a “cytokine storm,” and the magnitude of the cytokine storm is associated with disease severity [16]. Therefore, increased CRP and IL-6 might be related to the disease severity of COVID-19. In addition, CRP and IL-6 might directly accelerate protein degradation and indirectly influence important metabolic pathways to make patients frail [20], thereby causing patients to progress rapidly to severe or critical infection or even death. CRP and IL-6 might serve as independent factors prewarning the severe infection occurrence. However, considering that the understanding of COVID-19 is still insufficient and the detailed reasons for the role of these factors in COVID-19 patients is unclear, further studies are needed.

The widespread use of COVID-19 in the global population is currently the most rigorous health threat. Although most cases are mild, the apparently large number of infections poses a considerable burden to the national medical resources in affected countries. In addition, since the window of common to severe transition is short and the clinical outcomes of severe patients are extremely poor, an increasing number of lives have been lost due to delay of life support and treatment. Early warning biomarkers are urgently needed to assist with timely treatment and early prevention of disease aggravation. The nomogram model showed good agreement between prediction and actual probability in the primary cohort as well as the validation cohort. Patients who are high risk based on the nomogram model could be closely monitored for clinical index during disease course, and early intervention could be initiated to shorten the time interval between the onset of severe disease and treatment, which increases the survival possibility.

There are some limitations of our study. First, the sample size for nomogram validation was relatively small. More cases as well as a prospectively designed study are needed to further prove the accuracy of our nomogram model. Second, the phylogenetic analysis revealed three different variants of SARS-CoV-2 that might present distinct disease severity, whereas the virus variant was not differentiated in this study, which might influence the accuracy of the early warning model [21]. Third, since different centers were involved and the treatment concept in different centers might differ due to the relatively urgent medical situation, the clinical outcomes might vary. Furthermore, a comprehensive and in-depth understanding of COVID-19 is still lacking regarding the pathology and virus source; therefore, the current findings need supportive data from broader and global analyses.

In conclusion, the nomogram (including age, dyspnea, lymphocyte count, CRP and IL-6) objectively and accurately prewarns the severe disease occurrence out of common type COVID-19 patients. Our findings provide good evidence to accurately estimate changes in the patient’s condition, thereby aiding prevention and advance treatment.

## METHODS

### Study design and patients

Between February 19, 2020 and April 7, 2020, 1059 COVID-19 patients admitted to Guanggu District, Hubei Maternal and Child Health Hospital were analyzed in this study. All the clinical characteristics were collected in the incipient stage when all the patients were diagnosed as common type COVID-19. During the period from hospitalization to discharge, part of patients changed into severe type. All patients with laboratory-confirmed SARS-CoV-2 infection were diagnosed with COVID-19 according to the diagnostic criteria proposed in the 7th version of the Guidelines on the Diagnosis and Treatment of COVID-19 by the National Health Commission of China (available at: http://www.nhc.gov.cn/). The laboratory-confirmed SARS-CoV-2 infection was defined as a positive result of a real-time reverse transcriptase polymerase chain reaction (RT-PCR) assay of nasal-pharyngeal swab sample or/and a positive result of SARS-CoV-2 specific IgM antibody and IgG antibody in a serum sample. All 1059 COVID-19 patients formed the primary cohort, and all of them had complete clinical data, including basic characteristics and laboratory tests, which were used as a training dataset for constructing the disease progression early warning model, fitting the parameters and internal validation. Furthermore, a total of 123 COVID-19 patients admitted to Huoshenshan (Fire God Mountain) Hospital were enrolled in the study. All 123 patients also had a confirmed diagnosis of COVID-19, similar to the patients in the primary cohort, and served as the validation cohort in the study. The clinical data of these 123 patients were used as a test dataset for external validation to assess the performance of this early warning model.

### Clinical classification for COVID-19

Clinical classification for COVID-19 was performed according to the 7th version of the Guidelines on the Diagnosis and Treatment of COVID-19 by the National Health Commission of China, which were as follows: (1) Mild type: the clinical symptoms were mild with no abnormal radiological findings; (2) moderate type: fever and respiratory symptoms were presented with pneumonia on chest computed tomography; (3) severe type: one of the following conditions had to be met: (a) respiratory distress, respiratory rate ≥30 per min; (b) oxygen saturation on quiescent condition ≤93%; (c) partial pressure of oxygen in arterial blood/fraction of inspired oxygen (PaO_2_/FiO_2_) ≤300 mmHg (1 mmHg = 0.133 kPa); and (4) critical type: one of the following conditions had to be met: (a) respiratory failure occurred and mechanical ventilation was required; (b) shock occurred; (c) patients with other organ dysfunction needing intensive care unit (ICU) monitoring and treatment. In the present study, there were 808 common type patients and 251 severe type patients in the primary cohort; there were 90 common type patients and 33 severe type patients in the validation cohort.

### Data collection

Clinical data of patients were collected from the database of hospitals, which included the following: sex, age, weight, symptoms (dyspnea, fever, cough, fatigue), laboratory tests (leukocyte count, lymphocyte count, neutrophil count, monocyte count, c-reactive protein (CRP), alanine aminotransferase (ALT), aspartate aminotransferase (AST), alkaline phosphatase (ALP), total bilirubin (TBIL), procalcitonin (PCT), and interleukin 6 (IL-6).

### Statistical analysis

SPSS 22.0 statistical software (IBM, Chicago, Illinois, USA) was used to perform the descriptive analysis and the univariate/multivariate logistic regression analysis. In the descriptive analysis, continuous variables were switched to dichotomous variables, among which age and weight were processed as dichotomous variables by the median value, and the laboratory indexes were processed as dichotomous variables by the normal range of each index. In the logistic regression analysis, only variables with a *P* value <0.05 in the univariate logistic regression analysis were further included in the multivariate logistic regression analysis with the *Stepwise* method to construct the severe disease early warning model. The *rms* package in R X64 3.6.3 (http://cran.rstudio.com/bin/windows/base/) was used to formulate the nomogram based on the multivariate logistic regression model (Supplementary Material) [22]. The predictive accuracy and discriminative ability of the nomogram were determined by the concordance index (C-index) and calibration curve. The performance and accuracy of the established nomogram were assessed by receiver operating characteristic (ROC) curve by *pROC* package in R (Supplementary Material). The area under ROC (AUC) were determined. Decision curve analysis (DCA) based on the net benefit was depicted by the package of *ggDCA* in R (Supplementary Material). A *P* value <0.05 was considered statistically significant.

## Supplementary Materials

Supplementary Material
