# Supplementary material for: Severe versus common COVID-19: an early warning nomogram model
Source: Aging (Albany NY). 2022 Jan 17;14(2):544–56. doi: 10.18632/aging.203832 (PMC8833119; doi:10.18632/aging.203832)
Supplement: Supplementary Material [file aging-14-203832-s001.pdf]

SUPPLEMENTARY MATERIALS

Related computerized programs for nomogram with R

```
library (rms)
library (Hmisc)
library (grid)
library (lattice)
library (Formula)
library (ggplot2)
library (foreign)
library (pROC)
library (ggDCA)
library (verification)
```

For nomogram

```
rawdata<− read.csv("nomo.csv")
Y <− rawdata$Y
Age <− rawdata$Age
Dyspnea <− rawdata$Dyspnea
LMPH <− rawdata$LMPH
IL6 <− rawdata$IL6
CRP <− rawdata$CRP
dev = rawdata[rawdata$category = =0,]
vad = rawdata[rawdata$category = =1,]
ddist <− datadist(rawdata)
options (datadist = ‘ddist’)
modelC2 <− lrm(Y ~ Age + Dyspnea + CRP+ IL6 + LMPH, data = rawdata, x = TRUE, y = TRUE)
nom <− nomogram(modelC2, fun= function(x)1/(1+exp(-x)),fun.at=c(0.01,0.03,0.1,0.25,0.5,0.7,0.9,0.99),lp = F, funlabel = “Risk”)
plot (nom)
```

For resampling validation of nomogram

```
Validate (modelC2, method = “boot”, B = 1000, dxy = T)
```

For computing the C-Index

```
r <− rcorrcens(Surv(Y) ~ predict(modelC2), data = rawdata)
r
```

For calibration curve

```
cal <− calibrate(modelC2, method = ‘boot’, B=1000)
plot(cal,xlim = c(0,1.0), ylim = c(0,1.0))
```

For ROC curve

```
modelC2 <− lrm(Y ~ Age + Dyspnea + CRP+ IL6 + LMPH, data = rawdata, x = TRUE, y = TRUE)
predict <− predict(modelC2)
write.csv(predict, “predict1.csv”)
rawdata<− read.csv(“cal000.csv”)
Y <− rawdata$Y
predict <− rawdata$predict
roc(rawdata$Y, rawdata$predict, plot = TRUE, print.thres = TRUE, print.auc = TRUE)
```

For DCA curve

```
d <− dca (modelC2)
ggplot (d)
```
